# External Validation of Pediatric Pneumonia and Bronchiolitis Risk Scores to Predict Mortality in Children Hospitalized in Kenya: A Retrospective Cohort Study

**DOI:** 10.1093/infdis/jiaf377

**Published:** 2025-07-22

**Authors:** Becky Gordon, Joyce U Nyiro, Harish Nair, Zakariya Sheikh, Esther Katama, Charles N Agoti, Ruonan Pei, Heather Zar, Ting Shi

**Affiliations:** Usher Institute, Edinburgh Medical School, University of Edinburgh, Edinburgh, United Kingdom; Institute of Genetics and Cancer, University of Edinburgh, Edinburgh, United Kingdom; Epidemiology and Demography Department, Kenya Medical Research Institute-Wellcome Trust Research Programme, Kilifi, Kenya; Usher Institute, Edinburgh Medical School, University of Edinburgh, Edinburgh, United Kingdom; School of Public Health, Nanjing Medical University, Nanjing, China; Medical Research Council/University of the Witwatersrand Rural Public Health and Health Transitions Research Unit (Agincourt), School of Public Health, Faculty of Health Sciences, University of the Witwatersrand, Johannesburg, South Africa; Edinburgh Medical School, College of Medicine and Veterinary Medicine, University of Edinburgh, Edinburgh, United Kingdom; Epidemiology and Demography Department, Kenya Medical Research Institute-Wellcome Trust Research Programme, Kilifi, Kenya; Epidemiology and Demography Department, Kenya Medical Research Institute-Wellcome Trust Research Programme, Kilifi, Kenya; School of Public Health, Pwani University, Kilifi, Kenya; Usher Institute, Edinburgh Medical School, University of Edinburgh, Edinburgh, United Kingdom; Department of Paediatrics and Child Health, Red Cross War Memorial Children's Hospital, Cape Town, South Africa; South African Medical Research Council Unit on Child and Adolescent Health, University of Cape Town, Cape Town, South Africa; Usher Institute, Edinburgh Medical School, University of Edinburgh, Edinburgh, United Kingdom

**Keywords:** pneumonia, acute lower respiratory tract infections, risk stratification, risk scores

## Abstract

**Background:**

Acute lower respiratory tract infections (ALRIs) are a leading cause of pediatric mortality in low- and middle-income countries. In recent years, substantial research has been done to enhance risk stratification of children presenting with ALRIs, in a bid to improve health outcomes in resource-limited settings. We sought to analyze the performance of several pediatric ALRI risk scores in the prediction of mortality among children hospitalized with ALRIs in Kenya.

**Methods:**

We retrospectively analyzed the data of 2182 children aged 2–24 months who were admitted to Kilifi County Referral Hospital, Kenya with severe ALRIs between January 2015 and December 2024. We evaluated the performance of 6 ALRI risk scores (RISC [HIV-negative], mRISC, RISC-Malawi, PERCH, PREPARE, and ReSVinet) in this population. Additionally, we created and evaluated a modified version of the ReSVinet score by including nutrition status. Discrimination was assessed using the area under the receiver operating characteristic curve (AUROC).

**Results:**

The mid-upper arm circumference (MUAC) version of the RISC-Malawi score showed the highest discrimination for the outcome of in-hospital mortality (AUROC, 0.83; 95% confidence interval, .79–.86), whilst all other scores showed acceptable discrimination (AUROC, 0.70–0.79). The modification of ReSVinet to include nutrition status significantly improved its AUROC from 0.72 to 0.79.

**Conclusions:**

All risk scores showed at least fair performance in the prediction of in-hospital mortality within our dataset. The RISC-Malawi (MUAC) score appears to be the most promising candidate for future implementation; however, further research is needed to evaluate the calibration, feasibility, and clinical utility of these scores.

## Teaser

We assessed the discrimination of 6 risk scores in 2182 children aged 2–24 months hospitalized with acute lower respiratory tract infections in Kenya. All scores were at least fair at predicting in-hospital mortality, with 1 score marginally better than the others.

Acute lower respiratory tract infections (ALRIs), such as pneumonia and bronchiolitis, are a leading cause of pediatric mortality in low and middle-income countries (LMICs) [1]. In 2021, ALRIs were estimated to have caused the deaths of over 700 000 children aged under 5 years globally, accounting for 14% of all deaths in this age group [2]. In Kenya, ALRIs are the leading cause of mortality in children aged under 5 years outside of the neonatal period, leading to approximately 5000 deaths annually [2]. In addition, pediatric ALRIs are associated with an increased risk of long-term lung disease [3] and a substantial financial burden on households [4–6].

The World Health Organization (WHO) has produced guidelines on the diagnosis and management of pneumonia in children aged 2–59 months, most recently updated in 2024 [7]. An earlier version of these guidelines [8] has been summarized into a classification system [9] to advise healthcare workers when a child with pneumonia may require hospitalization.

In recent years, substantial research has sought to improve risk stratification of children presenting with ALRIs in LMICs. This effort aims to facilitate the early and accurate detection of children at high risk of mortality, who need close support, with the hope of improving outcomes while ensuring efficient allocation of often limited resources. We have identified 5 risk scores for the prediction of in-hospital mortality in children with pneumonia in LMICs: the Respiratory Index of Severity in Children (RISC) [10], modified-RISC (mRISC) [11], RISC-Malawi [12], Pneumonia Etiology Research for Child Health (PERCH) [13], and Pneumonia Research Partnership to Assess WHO Recommendations (PREPARE) [14] scores. Previous studies have externally validated the RISC (human immunodeficiency virus [HIV]-negative), RISC-Malawi, and PERCH scores [12, 15, 16]; however, no study has compared the performance of all scores within the same population.

In addition, a previous validation study in Rwanda found 2 bronchiolitis severity scores (Respiratory Syncytial Virus Network [ReSVinet] [17] and the Liverpool Infant Bronchiolitis Severity Score [LIBSS] [18]) showed excellent discrimination for in-hospital mortality in infants with respiratory illness (n = 100); notably, half of the included children were diagnosed with pneumonia [19]. Despite both scores being developed within high-income countries, these initial results suggest promise for their use in LMICs. Further validation and comparison to other ALRI risk scores within a larger population is warranted.

The primary aim of this study was to assess the discrimination of pediatric ALRI risk scores for the outcome of in-hospital mortality among young children admitted to hospital with ALRIs in Kilifi, Kenya.

## METHODS

### Risk Scores Under Study

We performed a scoping review to identify scores that had either been developed to predict mortality in children hospitalized with pneumonia in LMICs or had previously been externally validated for mortality prediction in the same population. We utilized a systematic review [20] as well as other relevant literature. We identified 5 pediatric pneumonia risk scores for the prediction of in-hospital mortality (RISC [10], mRISC [11], RISC-Malawi [12], PERCH [13], PREPARE [14]), which were developed using data from LMICs, and 2 bronchiolitis severity scores (ReSVinet [17] and LIBSS [18 ]), which had previously been validated in Rwanda for the prediction of in-hospital mortality [19]. We did not include LIBSS in our analysis, due to the large proportion of variables missing from our dataset (approximately 50%). Characteristics of each score's development dataset are summarized in Supplementary Table 1.

### Study Design

We retrospectively evaluated each score in our study population and assessed their discrimination for the outcome of in-hospital mortality. The study was reported in accordance with the TRIPOD guidelines [21] (Supplementary File 1).

### Study Population

The study population came from an ongoing inpatient respiratory pathogen surveillance study at Kilifi County Referral Hospital (KCRH), Kenya conducted by the Kenya Medical Research Institute-Wellcome research program; the study received ethical approval from their Scientific and Ethics Review Unit (protocol No. 3178). The data collection process has been described previously [22].

Briefly, data were collected from children aged 1 day to 59 months presenting to KCRH with symptoms of severe ALRI (history of cough or difficulty breathing, and 1 or more of the following: chest indrawing, oxygen saturations < 90%, unable to feed, prostrate, or unconscious). Upon admission, each child was assessed by a doctor or clinician using a standardized clinical history and examination, and a standard set of investigations. Primary and secondary discharge diagnoses and outcome data were recorded. Informed written consent was obtained from the child's caregiver for inclusion in the surveillance. For this study, we were provided with an anonymized version of the data collected over the previous 10 years (admissions occurring from 1 January 2015 to 31 December 2024) under a data sharing agreement.

For our main study population (cohort A), we only included children aged 2–24 months due to the different definition of pneumonia and treatment guidelines in children under 2 months [23], and to ensure all scores were being assessed within their intended age range. We excluded children without a primary or secondary discharge diagnosis of ALRI, defined here as pneumonia or bronchiolitis, to ensure our analysis was restricted to children whose respiratory presentation was due to ALRIs and not due to a severe manifestation of another condition, such as anemia or malaria.

Children were excluded from the analysis if they had an outcome other than discharge or death. Children were not excluded if they had a secondary health condition. A complete case analysis was undertaken, therefore children with missing data were excluded.

### Score Estimations

Supplementary Tables 2 and 3 summarize the parameters and weights used in each score. There are 2 versions of the RISC-Malawi score, using either mid-upper arm circumference (MUAC) or weight-for-age z-score (WAZ) as the measure of malnutrition [12]; we assessed both versions, and they are henceforth referred to as RISC-Malawi (MUAC) and RISC-Malawi (WAZ), respectively. The HIV-negative version of the RISC score was used due to the lack of information regarding patients' HIV status.

The components of each score were compared with the collected variables to estimate the total score for each child at admission. For any score components not directly available in our dataset, comparable variables were identified and utilized in their place. If such a comparable variable did not exist, the score was calculated without that component. The algorithms used for our evaluations are presented in Supplementary Tables 2 and 3.

### ReSVinet Score Modification

Malnutrition is recognized as a significant risk factor for mortality in children hospitalized with severe ALRI [24] and is a parameter in all assessed risk scores, apart from ReSVinet [10–14]. We decided to modify the ReSVinet score to see if incorporating nutrition status would increase the score's discrimination. In line with how ReSVinet scores symptom severity, we assigned 2 points to children with moderate malnutrition and 3 points to children with severe malnutrition. We compared the impact of using 3 different measures of malnutrition: MUAC [25–28], WAZ [28], and weight-for-length z-score (WLZ) [26–29]. For the z-scores, thresholds of < −2 and < −3 were used to classify moderate and severe malnutrition, whilst for MUAC, cutoffs of < 12.5 cm and < 11.5 cm were used [25, 28].

### Statistical Analysis

Descriptive statistics were produced for the main population, stratified by outcome. Frequency and percentage occurrence are presented for each variable. Univariable and multivariable logistic regression was used to calculate crude and adjusted (for age and sex) odds ratios.

The outcome of interest was in-hospital mortality. The discrimination of each score was assessed within the main population (cohort A), as well as stratified by age group, using the area under the receiver operating characteristic curve (AUROC). To guide our interpretation of the AUROC, we used the thresholds employed by Hosmer et al: < 0.50 implies no discrimination, 0.50–0.69 implies poor discrimination, 0.70–0.79 implies acceptable discrimination, 0.80–0.89 implies good discrimination, and ≥ 0.90 implies excellent discrimination [30]. DeLong's method was used to calculate confidence intervals (CIs) and perform 2-sided hypothesis tests between AUROCs [31]. A *P* value of < .01 was taken to be significant. Additionally, we calculated the sensitivity, specificity, and Youden J index at all possible thresholds, and define the “optimal” cutoff as that which maximizes the Youden J index.

Calibration was not assessed as most of the initial publications proposing the included risk scores provided insufficient information to allow for calculation of predicted probabilities (eg, omitting model intercept). Observed mortality for each score value and at each potential cutoff were calculated.

### Sensitivity Analysis

Sensitivity analyses were carried out by assessing score performance in all 2–24 month olds who presented with symptoms of severe ALRI (including those without a discharge diagnosis of ALRI) (cohort B), and in children aged 2–59 months (cohort C). We also assessed performance when including children with missing data (cohort D). For these children, the corresponding score components were skipped and the remainder of the score evaluated as normal. Additionally, we sought to explore the potential impacts of the coronavirus disease 2019 (COVID-19) pandemic by assessing score performance after excluding all admissions during the first part of pandemic, defined as the year following the first detected case in Kenya [32] (ie, 13 March 2020 to 13 March 2021) (cohort E), and by only including admissions occurring before the date of the first detected case (cohort F). The inclusion criteria for each sensitivity analysis cohort are summarized in Supplementary Table 4.

All statistical analysis was performed using R (version 4.4.1) [33] and RStudio [34]. All analysis code is available at https://github.com/rgordon482/ALRI-risk-score-val.

## RESULTS

### Data Characteristics

**Figure 1.**
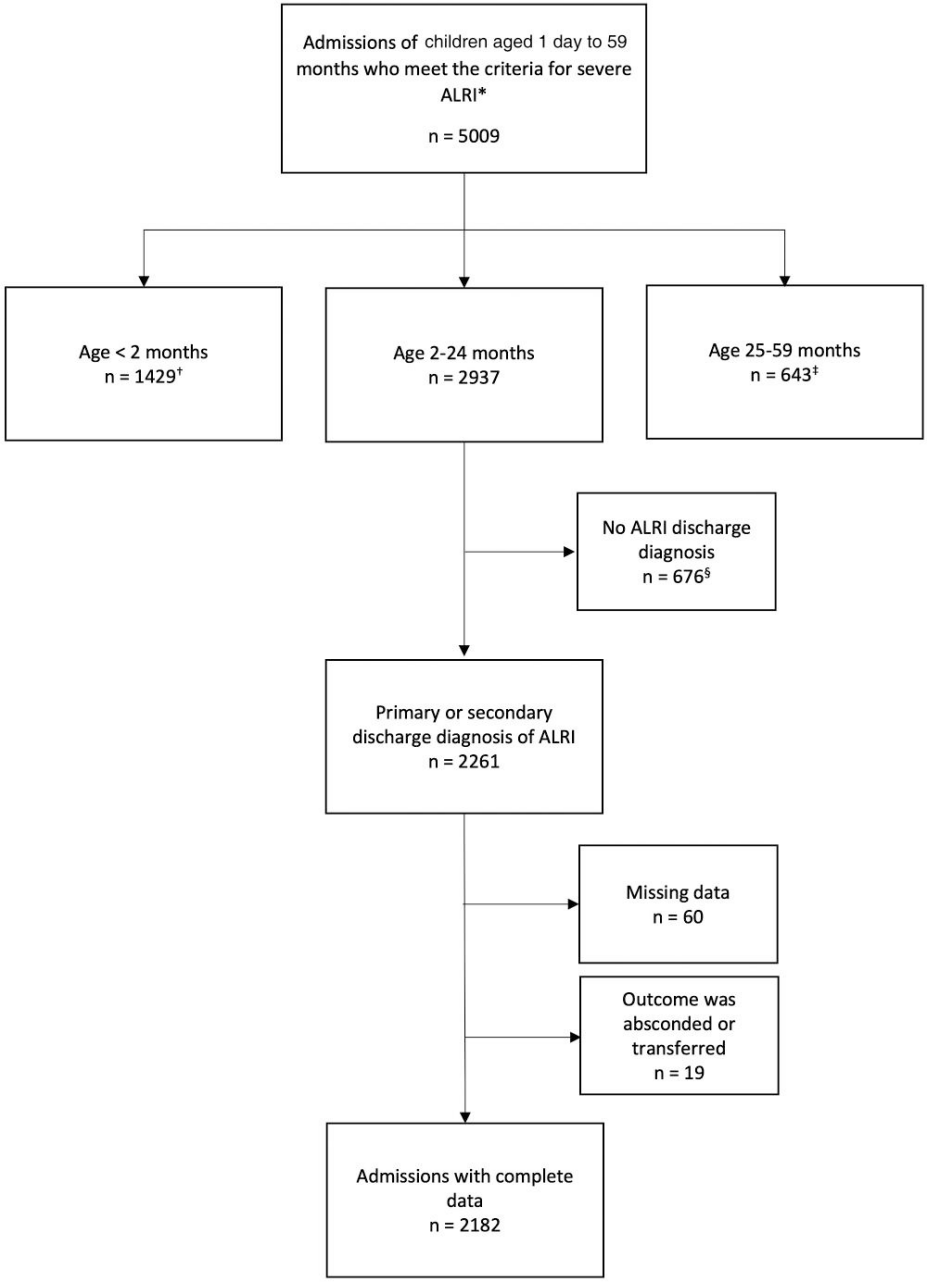
Flow chart of study participant inclusion. *History of cough or difficulty breathing and 1 or more of the following: chest indrawing, oxygen saturations < 90%, unable to feed, prostrate, or unconscious. †Of these admissions, 526 resulted in a primary or secondary discharge diagnosis of acute lower respiratory tract infection (ALRI). Of these, 94 had missing data and 3 had an outcome other than death or discharge. ‡Of these admissions, 346 resulted in a primary or secondary discharge diagnosis of ALRI. Of these, 9 had missing data and 3 had an outcome other than death or discharge. §Of these admissions, 27 had missing data and 15 had an outcome other than death or discharge.

Between January 2015 and December 2024, there were 2261 admissions of children aged 2–24 months who met the criteria for severe ALRI and had a discharge diagnosis of ALRI (Figure 1). Of these, 60 admissions were excluded due to missing data (4 admissions missing weight and associated z-scores, 16 missing only WAZ, 38 missing only WLZ, and 2 missing binary indicator to “vomits everything”) and 19 for having an outcome other than death or discharge (5 absconded and 14 were transferred). The primary study population (cohort A) therefore contained 2182 admissions with an in-hospital case fatality rate of 7.0% (n = 152). Table 1 presents descriptive statistics for cohort A stratified by outcome.

**Table 1.** Summary Statistics for the Study Population (N = 2182) Stratified by Outcome

Variable	All Patients, No. (%) (n = 2182)	Died In Hospital, No. (%) (n = 152)	Discharged, No. (%) (n = 2030)	Odds Ratio (95% CI)	Adjusted Odds Ratio (95% CI)
Age, mo					
2–5	863 (39.6)	80 (52.6)	783 (38.6)	Ref	Ref
6–12	766 (35.1)	44 (28.9)	722 (35.6)	0.6 (.4–.9)	0.6 (.4–.9)
13–24	553 (25.3)	28 (18.4)	525 (25.9)	0.5 (.3–.8)	0.5 (.3–.8)
Sex					
Female	934 (42.8)	78 (51.3)	856 (42.2)	Ref	Ref
Male	1248 (57.2)	74 (48.7)	1174 (57.8)	0.7 (.5–1.0)	0.7 (.5–1.0)
Respiratory rate					
< Age-specific cutoff^a^	759 (34.8)	46 (30.3)	713 (35.1)	Ref	Ref
0–9 bpm above age-specific cutoff	618 (28.3)	35 (23)	583 (28.7)	0.9 (.6–1.5)	1 (.6–1.5)
10–19 bpm above age-specific cutoff	465 (21.3)	30 (19.7)	435 (21.4)	1.1 (.7–1.7)	1.1 (.7–1.8)
> 20 bpm above age-specific cutoff	340 (15.6)	41 (27)	299 (14.7)	2.1 (1.4–3.3)	2.3 (1.5–3.7)
Mid-upper arm circumference, cm					
≥ 12.5	1316 (60.3)	37 (24.3)	1279 (63)	Ref	Ref
≥ 11.5 and < 12.5	401 (18.4)	29 (19.1)	372 (18.3)	2.7 (1.6–4.4)	2.6 (1.6–4.3)
< 11.5	465 (21.3)	86 (56.6)	379 (18.7)	7.8 (5.2–11.7)	7.4 (4.8–11.2)
Weight-for-age z-score					
≥ −2	1410 (64.6)	63 (41.4)	1347 (66.4)	Ref	Ref
≥ −3 and < −2	418 (19.2)	35 (23)	383 (18.9)	2.0 (1.3–3.0)	2.5 (1.6–3.8)
< −3	354 (16.2)	54 (35.5)	300 (14.8)	3.8 (2.6–5.7)	5.3 (3.5–8.0)
Weight-for-length z-score					
≥ −2	1763 (80.8)	101 (66.4)	1662 (81.9)	Ref	Ref
≥ −3 and < −2	300 (13.7)	36 (23.7)	264 (13)	2.2 (1.5–3.4)	2.9 (1.9–4.4)
< −3	119 (5.5)	15 (9.9)	104 (5.1)	2.4 (1.3–4.2)	3.5 (1.9–6.4)
Conscious level					
Normal	1583 (72.5)	58 (38.2)	1525 (75.1)	Ref	Ref
Agitated	67 (3.1)	14 (9.2)	53 (2.6)	6.9 (3.6–13.2)	7.1 (3.7–13.5)
Lethargic	335 (15.4)	30 (19.7)	305 (15)	2.6 (1.6–4.1)	2.8 (1.8–4.4)
Prostrate	163 (7.5)	36 (23.7)	127 (6.3)	7.5 (4.7–11.7)	7.3 (4.6–11.5)
Unconscious	34 (1.6)	14 (9.2)	20 (1)	18.4 (8.9–38.3)	19.4 (9.2–41.0)
Oxygen saturations					
> 92%	1778 (81.5)	78 (51.3)	1700 (83.7)	Ref	Ref
90%–92%	120 (5.5)	8 (5.3)	112 (5.5)	1.6 (.7–3.3)	1.5 (.7–3.2)
< 90%	284 (13)	66 (43.4)	218 (10.7)	6.6 (4.6–9.4)	6.4 (4.5–9.1)
Presence of prespecified signs or symptoms on initial presentation					
Chest indrawing	2144 (98.3)	143 (94.1)	2001 (98.6)	0.2 (.1–.5)	0.2 (.1–.4)
Convulsion	90 (4.1)	13 (8.6)	77 (3.8)	2.4 (1.3–4.4)	2.5 (1.3–4.6)
Cough	2054 (94.1)	129 (84.9)	1925 (94.8)	0.3 (.2–.5)	0.3 (.2–.5)
Crackles	1133 (51.9)	71 (46.7)	1062 (52.3)	0.8 (.6–1.1)	0.8 (.6–1.1)
Cyanosis	29 (1.3)	12 (7.9)	17 (0.8)	10.1 (4.8–21.7)	10.1 (4.7–21.7)
Decreased skin turgor	37 (1.7)	13 (8.6)	24 (1.2)	7.8 (3.9–15.7)	8.8 (4.3–17.9)
Deep breathing	480 (22)	70 (46.1)	410 (20.2)	3.4 (2.4–4.7)	3.4 (2.4–4.8)
Diarrhea	309 (14.2)	27 (17.8)	282 (13.9)	1.3 (.9–2.1)	1.5 (.9–2.3)
Head nodding	375 (17.2)	28 (18.4)	347 (17.1)	1.1 (.7–1.7)	1.0 (.7–1.6)
Nasal flaring	1432 (65.6)	105 (69.1)	1327 (65.4)	1.2 (.8–1.7)	1.2 (.8–1.7)
Pallor	387 (17.7)	59 (38.8)	328 (16.2)	3.3 (2.3–4.7)	3.6 (2.5–5.2)
Sunken eye	84 (3.8)	18 (11.8)	66 (3.3)	4.0 (2.3–6.9)	4.5 (2.6–7.9)
Vomiting	434 (19.9)	36 (23.7)	398 (19.6)	1.3 (.9–1.9)	1.4 (.9–2.1)
Vomits everything	127 (5.8)	17 (11.2)	110 (5.4)	2.2 (1.3–3.8)	2.6 (1.5–4.4)
Unable to drink	244 (11.2)	42 (27.6)	202 (10)	3.5 (2.4–5.1)	3.2 (2.2–4.7)
Wheeze	372 (17)	19 (12.5)	353 (17.4)	0.7 (.4–1.1)	0.7 (.4–1.2)

Abbreviations: bpm, breaths per minute; CI, confidence interval; Ref, reference.

^a^World Health Organization cutoff for fast breathing is 40 bpm for children aged 2–11 mo and 50 bpm for children 12–24 mo [9].

### Score Discrimination

Table 2 displays the AUROC of each score for the outcome of in-hospital mortality in cohort A, and Supplementary Table 5 presents the associated *P* value matrix. RISC-Malawi (MUAC) showed good discrimination, whilst all other scores showed acceptable discrimination. There were statistically significant differences between the AUROCs of RISC-Malawi (MUAC) and all other scores. The PREPARE and PERCH scores also showed evidence of a significant difference in AUROC with the RISC score. No other significant differences were observed.

**Table 2.** AUROC of Each Score for In-Hospital Mortality

Score	AUROC for In-Hospital Mortality (95% CI)
mRISC	0.76 (.72–.80)
PERCH	0.77 (.73–.81)
PREPARE	0.79 (.75–.82)
ReSVinet	0.72 (.67–.76)
RISC	0.70 (.66–.75)
RISC-Malawi (MUAC)	0.83 (.79–.86)
RISC-Malawi (WAZ)	0.78 (.73–.82)

Abbreviations: AUROC, area under the receiver operating characteristic curve; CI, confidence interval; mRISC, modified Respiratory Index of Severity in Children; MUAC, mid-upper arm circumference; PERCH, Pneumonia Etiology Research for Child Health; PREPARE, Pneumonia Research Partnership to Assess WHO Recommendations; ReSVinet, Respiratory Syncytial Virus Network; RISC, Respiratory Index of Severity in Children; WAZ, weight-for-age z-score.

Table 3 presents the sensitivity, specificity, and case fatality rate at each score's optimal threshold. RISC-Malawi (MUAC) had the highest Youden J index of all scores, with its optimal high-risk cutoff being ≥ 7 points. At this threshold, RISC-Malawi (MUAC) had a sensitivity of 82.2% and a specificity of 71.5%. Depending on the intended use of the score and the resource limitations of the setting, different high-risk thresholds may be appropriate. Supplementary Tables 6–12 contain the sensitivity, specificity, and case fatality rate of each score at every threshold, as well as the scoring distributions. Supplementary Tables 13 and 14 present the AUROCs for each score stratified by age group, and the associated *P* value matrix. There were no statistically significant differences in AUROCs between age groups. Supplementary Tables 15–19 show the results of the sensitivity analyses for age, discharge diagnosis, missing data, admissions which took place before the COVID-19 pandemic, and admissions which took place outside of the first year of the COVID-19 pandemic. No significant differences were found in comparison to the main sample results (*P* values ≥ .01).

**Table 3.** Sensitivity and Specificity of Each Score at Optimal Threshold

Score	Cutoff for High Risk	Sensitivity, %	Specificity, %	Proportion of Population Classed as High Risk, %	Youden J Index	Observed Case Fatality Rate for High Risk, %
mRISC	≥ 4	61.8	78.2	24.6	0.400	17.5
PERCH	≥ 3	71.7	74.6	28.6	0.463	17.5
PREPARE	≥ 5	63.2	82.3	20.9	0.455	21.1
ReSVinet	≥ 9	56.6	77.7	24.7	0.343	16.0
ReSVinet + Nutrition (MUAC)	≥ 10	65.8	76.5	26.4	0.423	17.3
RISC	≥ 3	72.4	59.0	43.2	0.314	11.7
RISC-Malawi (MUAC)	≥ 7	82.2	71.5	32.3	0.537	17.8
RISC-Malawi (WAZ)	≥ 5	73.0	73.0	30.2	0.460	16.8

Abbreviations: mRISC, modified Respiratory Index of Severity in Children; MUAC, mid-upper arm circumference; PERCH, Pneumonia Etiology Research for Child Health; PREPARE, Pneumonia Research Partnership to Assess WHO Recommendations; ReSVinet, Respiratory Syncytial Virus Network; RISC, Respiratory Index of Severity in Children; WAZ, weight-for-age z-score.

### Modification of the ReSVinet Score

Supplementary Tables 20 and 21 present the AUROCs of the modified ReSVinet scores in cohort A and the associated *P* value matrix. The inclusion of MUAC as the malnutrition measure resulted in the largest increase in AUROC from the original ReSVinet score and provided a significant increase in comparison to the scores modified using WAZ or WLZ. MUAC was therefore used in the final modified score (henceforth referred to as the modified ReSVinet score).

The modified ReSVinet score had an AUROC of 0.79 (95% CI, .76–.83) for in-hospital mortality. Its optimal cutoff was ≥ 10 which corresponded to a sensitivity of 65.8% and a specificity of 76.5%. Supplementary Table 22 presents the scoring distribution of the modified score, along with its sensitivity and specificity at each threshold.

The modified ReSVinet score performed significantly better than the original ReSVinet score and the RISC score (*P* values < .01); however, no significant differences were found with any other score (Supplementary Table 5).

## DISCUSSION

We assessed the performance of 5 existing pneumonia risk scores and 1 bronchiolitis severity score in the prediction of mortality in children admitted to KCRH, Kenya with severe ALRIs. The RISC-Malawi (MUAC) score showed good discrimination (AUROC = 0.83; 95% CI, .79–.86), whilst all other scores showed acceptable discrimination (AUROC range, 0.70–0.79). All scores other than RISC-Malawi (MUAC) showed relatively low sensitivity (≤ 73.0%) at their optimal threshold. The low sensitivity of these scores suggests that their implementation could result in a significant number of children who are at high risk of mortality being misclassified as low risk. The threshold of these scores could be altered to increase their sensitivity; however, their specificity would suffer as a result. RISC-Malawi (MUAC) displayed the highest Youden J index of all scores (Youden J = 0.537 at a high-risk cutoff of ≥ 7), resulting in an optimal cutoff with a high sensitivity (82.2%) whilst maintaining a good specificity (71.5%). Nevertheless, we would recommend further external validation studies to replicate our findings before any future implementation.

Modifying the ReSVinet score by adding nutritional status as a parameter significantly improved its discrimination in our population, with an increase in AUROC from 0.72 to 0.79. The RISC-Malawi (MUAC) score showed a significant improvement in discrimination over all other scores, except the modified ReSVinet score, including the RISC-Malawi (WAZ) score. The superior performance of RISC-Malawi (MUAC) compared to RISC-Malawi (WAZ), and the finding that MUAC was the optimal malnutrition measure in our modification of ReSVinet, is consistent with previous work indicating the greater prognostic ability of MUAC over other malnutrition measures [35]. Further research may be warranted into the predictive power of low MUAC for mortality in young children with severe ALRI.

All assessed pneumonia risk scores were generated using multivariable logistic regression, followed by scaling and rounding of the coefficients to create a simplified risk score. Different scalars were used across the developed scores, with RISC-Malawi using the largest values. This may have resulted in improved performance, as lower scalar values are associated with an increased loss of information when coefficients are rounded [36]. Future studies may wish to assess the impact of scalar choice on score performance.

Before development of any new ALRI risk scores, researchers should first examine the evidence base on existing scores to assess their appropriateness for clinical use, and hence whether there is a need for new scores. To maximize the performance of future scores, researchers should ensure development datasets are feature rich to allow for the testing of as many score components as possible.

Scores should be simplified to maximize feasibility within a clinical setting; however, care should be taken to ensure scalar choice does not significantly decrease model performance. Additionally, all research should utilize the TRIPOD checklist or similar to ensure adequate reporting to enable full external validation, including assessment of calibration [21]. Beyond external validation of discrimination and calibration, future work is required to assess the reliability, feasibility, and utility of these risk scores in clinical practice.

Several studies have previously externally validated the performance of the assessed scores in children hospitalized with ALRIs across various LMICs. Ogero et al found the RISC-Malawi (MUAC) score had acceptable performance within a large cross-hospital dataset in Kenya [37]. Rees et al found that both versions of the RISC-Malawi score showed fair discrimination when assessed within subsets of the PREPARE dataset [15]. It is notable that each score was assessed in a different subsample with sample size varying significantly, potentially inhibiting valid comparisons. Rees et al also assessed the performance of the PERCH score in a subset of the PREPARE dataset, where it showed poor performance [15]. They note their analysis was limited by low sample size (n = 732) and unavailability of key variables, potentially explaining the decreased performance in comparison to our results.

Several external validation studies have been carried out for the RISC (HIV-negative) score, with performance varying significantly [12, 15, 16]. Our results indicate this score shows acceptable discrimination, which is in line with a previous external validation in Malawi by Hooli et al [12]. The RISC (HIV-negative) score was also assessed in a subset of the PREPARE dataset [15], showing poor performance; however, the reported AUROC (0.66; 95% CI, .58–.73) was not significantly different from that found in our analysis. The RISC (HIV-negative) score showed excellent discrimination for in-hospital mortality in a small sample of children hospitalized with ALRI in India [16]; however, these results may have been affected by the low sample size (n = 180).

A previous external validation of the ReSVinet score in Rwanda [19] showed excellent discrimination for in-hospital mortality; however, this study was limited by a low sample size (n = 100). The improved performance in comparison to our results could also be due to their contemporaneous evaluation of the ReSVinet score by health professionals treating the child, compared to our retrospective, algorithmic evaluation.

Our study contains several limitations. First, we only had data for children admitted to hospital. We had no data on children who presented to hospital but were not admitted, or who did not present. Moreover, there may have been temporal changes in admission criteria, for example, due to service pressures or resource limitations. Also, data were missing between March 2020 and June 2020 due to interruptions caused by the COVID-19 pandemic.

Second, we were only able to evaluate 3 out of the 6 scores in full, with some scores missing up to a third of their components in our evaluation. For the ReSVinet score, we were unable to assess the components relating to apnea or medical intervention, and we had insufficient data to assess for mild severity presentations of feeding intolerance, general condition, and tachypnea. For the PERCH score, we were unable to assess the grunting or illness duration components, whilst for the mRISC score we were unable to assess the night sweats component or the 2 components that required information regarding laboratory-confirmed malaria. Much of this missingness may be due to the use of signs/symptoms not routinely collected in clinical practice as score parameters. We did not evaluate the LIBSS score, due to at least half of its components being missing in our dataset, and all other scores were assessed in the absence of any missing components. This may have affected the discrimination of these scores in comparison to their full use as intended. Another potential limitation is the interpretation of scoring components. Due to ReSVinet's parameters being more open to interpretation than other scores (rating presentation severity rather than the presence or absence of specific symptoms), an evaluation algorithm was formed using the component descriptors and clinical input from HN. Our algorithmic approach restricted our ability to fully represent the granularity of each component, and may not align with the interpretation of healthcare workers using the score.

Third, we did not account for the treatment received by each child. It is possible, due to changes in guidelines or resource limitations, that children received different treatments, including oxygen, for the same presentation, acting as a confounder in our analysis. We also had no information regarding children's HIV status and therefore may have inadvertently used the RISC (HIV-negative) score in children with HIV.

Fourth, our primary analysis (cohort A) only included children with a discharge diagnosis of ALRI, to reduce confounding from other causes of respiratory difficulty. However, this may have wrongfully excluded children with ALRI who were not coded as such. To explore this, we conducted a sensitivity analysis assessing score performance in all children presenting with symptoms of severe ALRI regardless of discharge diagnosis (cohort B). This showed no statistically significant differences (Supplementary Table 15).

## CONCLUSION

Our study presents the simultaneous external validation of 6 pediatric ALRI risk/severity scores in Kenya, along with the modification of an existing bronchiolitis severity score, which improved its discrimination within our study population. Assessing all scores within the same population enabled us to carry out the most comprehensive comparison of these risk scores to date, with all showing at least fair discrimination for in-hospital mortality. The RISC-Malawi (MUAC) score appears most promising for the discrimination of patients at high risk of in-hospital mortality, potentially owing to its use of MUAC as a malnutrition indicator. Further investigation into the use of MUAC as a predictor of ALRI-related in-hospital mortality may be warranted.

Further research should investigate the feasibility and utility of these scores in clinical practice. This work is imperative to understand the challenges of introducing such scores, and to understand whether they can assist in the effective allocation of resources and ultimately decrease adverse outcomes, such as mortality.

## Supplementary Material

jiaf377_Supplementary_Data

## Supplementary Data

Supplementary materials are available at *The Journal of Infectious Diseases*  online (http://jid.oxfordjournals.org/). Supplementary materials consist of data provided by the author that are published to benefit the reader. The posted materials are not copyedited. The contents of all supplementary data are the sole responsibility of the authors. Questions or messages regarding errors should be addressed to the author.

## Notes

***Author contributions.*** T. S., with input from B. G. and J. N., conceptualized the study. E. K., C. N. A., and J. N. contributed to data collection. B. G., with input from Z. S. and R. P., led data analysis. B. G., with support from R. P., wrote the codes for analysis. T. S., H. N., J. N., E. K., C. N. A., Z. S., H. Z., and B. G. led the data interpretation. B. G., with input from Z. S., wrote the first draft of the manuscript. All authors contributed to data interpretation and critically reviewed the manuscript, and read and approved the final draft of the manuscript. B. G. and T. S. had full access to and verified all the study data. All authors had final responsibility for the decision to submit for publication.

***Data availability.*** We do not have a protocol. The dataset used in this analysis is not publicly available, as it contains sensitive personal data. Data access requests can be made to the Data Governance Committee (dgc@kemri-wellcome.org).

***Financial support.*** BG acknowledges the receipt of studentship awards from the Health Data Research UK-The Alan Turing Institute Wellcome PhD Programme in Health Data Science (Grant Ref: 218529/Z/19/Z).
