# Supplementary material for: External Validation of Pediatric Pneumonia and Bronchiolitis Risk Scores to Predict Mortality in Children Hospitalized in Kenya: A Retrospective Cohort Study
Source: J Infect Dis. 2025 Jul 22;233(1):e230–8. doi: 10.1093/infdis/jiaf377 (PMC12811851; doi:10.1093/infdis/jiaf377)
Supplement: jiaf377_Supplementary_Data [file jiaf377_supplementary_data.zip › Supplementary_tables.docx]

# Supplementary tables for “External validation of paediatric pneumonia and bronchiolitis risk scores to predict mortality in children hospitalised with ALRI in coastal Kenya: a retrospective cohort study”

## Becky Gordon^1,2^, Zakariya Sheikh^6^, Joyce U. Nyiro^3^, Harish Nair^1,4,5^, Esther Katama^3^, Charles N. Agoti^3,7^, Ruonan Pei^1^, Heather Zar^8^, Ting Shi^1^

1. Usher Institute, Edinburgh Medical School, University of Edinburgh, Scotland
2. Institute of Genetics and Cancer, University of Edinburgh, Edinburgh, Scotland
3. Epidemiology and Demography Department, KEMRI-Wellcome Trust Research Programme, Kilifi, Kenya
4. School of Public Health, Nanjing Medical University, Nanjing, China
5. MRC/Wits Rural Public Health and Health Transitions Research Unit (Agincourt), School of Public Health, Faculty of Health Sciences, University of the Witwatersrand, Johannesburg, South Africa
6. Edinburgh Medical School, College of Medicine and Veterinary Medicine, University of Edinburgh, Edinburgh, Scotland
7. Pwani University, Kenya
8. Department of Paediatrics and Child Health, Red Cross War Memorial Children's Hospital, Cape Town 7700, South Africa; South African Medical Research Council Unit on Child and Adolescent Health, University of Cape Town, Cape Town, South Africa

**Correspondence to**:

Dr. Ting Shi

Usher Institute,

University of Edinburgh

Edinburgh

EH16 4UX UK

Ting.shi@ed.ac.uk

## List of tables

[Supplementary table 1: Development dataset summaries of included risk scores 3](#_Toc196312034)

[Supplementary table 2: ReSVinet score evaluation algorithm. For each score component, select the highest point value for which the child meets the descriptor. 5](#_Toc196312035)

[Supplementary table 3: Score evaluation algorithms for assessed pneumonia risk scores. 9](#_Toc196312036)

[Supplementary table 4: Inclusion criteria for main cohort (cohort A) and all cohorts used for sensitivity analysis (cohorts B-F). 12](#_Toc196312037)

[Supplementary table 5: P-value matrix from pairwise AUROC testing of scores evaluated in cohort A. 13](#_Toc196312038)

[Supplementary table 6: Score distribution for the mRISC score and sensitivity/specificity at each cut-off. 14](#_Toc196312039)

[Supplementary table 7: Score distribution for the PERCH score and sensitivity/specificity at each cut-off. 15](#_Toc196312040)

[Supplementary table 8: Score distribution for the PREPARE score and sensitivity/specificity at each cut-off. 16](#_Toc196312041)

[Supplementary table 9: Score distribution for the ReSVinet score and sensitivity/specificity at each cut-off. 17](#_Toc196312042)

[Supplementary table 10: Score distribution for RISC (HIV-Negative) score and sensitivity/specificity at each cut-off. 18](#_Toc196312043)

[Supplementary table 11: Score distribution for RISC-Malawi (MUAC) score and sensitivity/specificity at each cut-off. 19](#_Toc196312044)

[Supplementary table 12: Score distribution for RISC-Malawi (WAZ) score and sensitivity/specificity at each cut-off. 21](#_Toc196312045)

[Supplementary table 13: AUROCs for in-hospital mortality, stratified by age group. 23](#_Toc196312046)

[Supplementary table 14: p-values of unpaired AUROC tests for age stratification. 23](#_Toc196312047)

[Supplementary table 15: AUROCs for in-hospital mortality, for scores assessed in all admissions 2-24 months regardless of discharge diagnosis (Cohort B) 24](#_Toc196312048)

[Supplementary table 16: AUROCs for in-hospital mortality, for scores assessed in admissions for children 2-59 months with a primary or secondary discharge diagnosis of ALRI (Cohort C) 24](#_Toc196312049)

[Supplementary table 17: AUROCs for in-hospital mortality, including children with missing data (Cohort D) 25](#_Toc196312050)

[Supplementary table 18: AUROCs for in-hospital mortality with admissions occurring in the year following the first case of COVID-19 excluded (i.e. Patients admitted between 13th March 2020 and 13th March 2021 (inclusive) not included). (Cohort E) 25](#_Toc196312051)

[Supplementary table 19: AUROCs for in-hospital mortality, excluding admissions post-COVID (i.e. excluding admissions on or after 13/03/20). (Cohort F) 26](#_Toc196312052)

[Supplementary table 20: AUROCs for in-hospital mortality for modified ReSVinet score, using either mid-upper arm circumference (MUAC), weight-for-length z-score (WLZ) or weight-for-age z-score (WAZ) as the malnutrition indicator. 27](#_Toc196312053)

[Supplementary table 21: P-value matrix from pairwise testing of modified ReSVinet scores. 27](#_Toc196312054)

[Supplementary table 22: Score distribution for the ReSVinet + Nutrition (MUAC) score and sensitivity/specificity at each cut-off. 28](#_Toc196312055)

Supplementary table 1: Development dataset summaries of included risk scores

|  | **ReSVinet**  [1] | **RISC [HIV-negative]** [2] | **mRISC** [3] | **RISC-Malawi*** [4] | **PERCH** [5] | **PREPARE** [6] |
| --- | --- | --- | --- | --- | --- | --- |
| Development method | Systematic review of respiratory severity scores + review/refinement with 90 clinicians. | Multivariable logistic regression | | | | |
| **Development dataset characteristics** | | | | | | |
| Location | - | South Africa | Kenya | Malawi | Various | Various |
| Time period | - | 1998 - 2001 | 2009-2012 | 2011-2014 | 2011-2012 | 1994-2014 |
| Age range | - | <24 months | <5 years | 2-59 months | 1-59 months | 2-59 months |
| Case inclusion criteria | - | Secondary analysis of vaccine trial data. Children admitted to hospital with LRTI during follow-up period. | Hospitalised with severe acute respiratory illness (SARI) (defined as acute onset of cough or difficulty breathing within the last 14 days requiring hospitalisation) | Hospitalised with pneumonia (defined using Malawi Ministry of Health Case Management Guidelines) | Hospitalised with severe or very severe pneumonia (2005 WHO definition) | Hospitalised with suspected pneumonia (2013 WHO definition) [Note criteria seems to vary by study] |
| Outcome of interest | Severity of bronchiolitis | In-hospital mortality | In-hospital mortality | In-hospital mortality | In-hospital mortality or mortality within 7 days of discharge | In-hospital mortality |
| Sample size | - | 2646 hospitalizations (including repeat hospitalizations) | 3581 | 14665 | 1802 | 27388 |
| Number of deaths (Case fatality rate) | - | 33 (1.3%) | 218 (6.1%) | 464 (3.2%) | 120 (6.7%) | 856 (3.1%) |

* Development dataset is the same for both versions of the RISC-Malawi score

Supplementary table 2: ReSVinet score evaluation algorithm. For each score component, select the highest point value for which the child meets the descriptor.

| **Score component/descriptor** | **Points** | **Evaluation algorithm for our dataset** |
| --- | --- | --- |
| **Feeding intolerance** |  |  |
| **Mild** Decreased appetite and/or isolated vomits with cough. | +1 | **Unavailable** |
| **Partial** Frequent vomits with cough, rejected feed but able to tolerate fluids sufficiently to ensure hydration. | +2 | Vomiting = “yes” |
| **Total** Oral/ intolerance or absolute rejection of oral feed, not able to guarantee adequate hydration orally. Required nasogastric and/or intravenous fluids | +3 | Vomits everything = “yes”  **OR**  Unable to drink = “yes” |
| **Respiratory difficulty** |  |  |
| **Mild** Not in basal situation but does not appear severe. Wheezing only audible with stethoscope, good air entrance. | +1 | Wheeze = “yes” |
| **Moderate** Makes some extra respiratory effort (intercostal and/or tracheosternal retraction). Presented expiratory wheezing audible even without stethoscope, and air entrance may be decreased in localized areas. | +2 | Indrawing = “yes”  OR  Flaring = “yes” |
| **Severe** Respiratory effort is obvious. Inspiratory and expiratory wheezing and/or clearly decreased air entry. | +3 | Head nodding = “yes”  OR  Cyanosis = “yes”  OR  Deep breathing = “yes” |
| **General condition/appearance** |  |  |
| **Mild** Not in basal situation, child was mildly uncomfortable but does not appear to be in a severe condition, not impress of severity. Parents are not alarmed. Could wait in the waiting room or even stay at home. | +1 | **Unavailable** |
| **Moderate** Patient looks ill, and will need medical exam and eventually further complementary exams and/ or therapy. Parents are concerned. Cannot wait in the waiting room. | +2 | Conscious level = “Agitated”  OR  Conscious level = “Lethargic” |
| **Severe** Agitated, apathetic, lethargic. No need of medical training to realize severity. Parents are very concerned. Immediate medical evaluation and/or intervention were required. | +3 | Conscious level = “Unconscious”  OR  Conscious level = “Prostrate”  OR  Convulsions = “yes” |
| **Fever** |  |  |
| **Yes, mild**  Central T ≥ 38 C and < 38.5 C | +1 | Axillary temperature ≥ 37.5 C AND < 38 C |
| **Yes, moderate**  Central T ≥ 38.5 C | +2 | Axillary temperature ≥ 38 C |
| **Respiratory rate** |  |  |
| **Normal**  < 2 m: 40–50 bpm,  2–6 m: 35–45 bpm,  6-12m: 30–40 bpm,  12-24m:25–35 bpm,  24-36m: 20–30 bpm | +0 | - |
| **Mild or occasional tachypnea** Presented episodes of tachypnea, well tolerated, limited in time by self-resolution or response to secretion aspiration or nebulization. | +1 | **Unavailable** |
| **Prolonged or recurrent tachypnea** Tachypnea persisted or recurred despite secretion aspiration and/or nebulization with bronchodilators. | +2 | Respiratory rate for age:  < 2 m: 50-70 bpm,  2–5m: 45-60 bpm,  6-11m: 40-55 bpm,  12-23m: 35-50 bpm,  24-36m: 30-40 bpm |
| **Severe alteration** Severe and sustained tachypnea. Very superficial and quick breath rate. Normal/low breath rate with obvious increased respiratory effort and/or mental status affected. Orientative rates of severe tachypnea:  < 2 m: > 70 bpm,  2–6 m: > 60 bpm,  6-12m: >55 bpm,  12-24m: >50 bpm,  24-36m: >40 bpm | +3 | Respiratory rate for age:  < 2 m: > 70 bpm,  2–5 m: > 60 bpm,  6-11m: >55 bpm,  12-23m: >50 bpm,  24-36m: >40 bpm, |
| **Apnoea** |  |  |
| **Yes** At least one episode of respiratory pause medically documented or strongly suggested through anamnesis. | +3 | **Unavailable** |
| **Medical intervention** |  |  |
| **Basic** Nasal secretions aspiration, physical examination, trial of nebulized bronchodilators, antipyretics. | +1 | **Unavailable** |
| **Intermediate** Oxygen therapy required. Complementary exams were needed (chest X-rays, blood gases, hematimetry. . .). Maintained nebulized therapy with bronchodilators. | +2 | **Unavailable** |
| **High** Required respiratory support with positive pressure (either non-invasive in CPAP, BiPAP or high-flow O2; or invasive through endotracheal tube). | +3 | **Unavailable** |

Supplementary table 3: Score evaluation algorithms for assessed pneumonia risk scores.

|  | **Points** | **Corresponding variables in our dataset** |
| --- | --- | --- |
| **mRISC** |  |  |
| Lab confirmed malaria | -1 | **Unavailable** |
| Weight-for-age z-score ≤ -2 | +1 | Weight-for-age z-score ≤ -2 |
| Dehydration | +1 | Decreased skin turgor = “yes”  OR  Sunken eyes = “yes” |
| Was unconscious | +1 | Conscious level = “Unconscious”  OR  Conscious level = “Prostrate” |
| Unable to drink/breastfeed | +1 | Unable to drink = “yes” |
| Night sweats | -1 | **Unavailable** |
| Chest wall in-drawing | +1 | Chest indrawing = “yes” |
| Lab confirmed malaria AND chest wall in-drawing | +1 | **Unavailable** |
| A.V.P.U scale – Not alert | +2 | Blantyre Coma Scale < 5 |
| **PERCH** |  |  |
| Age 1-11 months | +2 | Age 1-11 months |
| Female | +1 | Female |
| Unresponsive but no deep breathing | +2 | (Conscious level = “Prostrate” OR Conscious level = “Unconscious”)  AND  Deep breathing = “no” |
| Unresponsive and deep breathing | +5 | (Conscious level = “Prostrate” OR Conscious level = “Unconscious”)  AND  Deep breathing = “yes” |
| Cough (observed) | -1 | Cough = “yes” |
| Grunting (observed) | +2 | **Unavailable** |
| Hypoxemia (Oxygen saturations < 92%) | +2 | Oxygen saturations < 92% |
| Maximum duration of illness > 2 days | +2 | **Unavailable** |
| Weight-for-height z-score < -3 | +3 | Weight-for-height z-score < -3 |
| Weight-for-height z-score < -2 AND ≥ -3 | +2 | Weight-for-height z-score < -2 AND ≥ -3 |
| **PREPARE** |  |  |
| Age 2-5 months | +2 | Age 2-5 months |
| Age 6-11 months | +1 | Age 6-11 months |
| Female | +1 | Female |
| Weight for age z-score < -3 | +3 | Weight for age z-score < -3 |
| Weight for age z-score -2 to -3 | +2 | Weight for age z-score -2 to -3 |
| Body temperature < 35.5C | +3 | Axillary temperature < 35.5C |
| Respiratory rate ≥ 20 breaths/min above age-specific cut-off  (Cut-offs: ≥ 50 breaths/min for children 2-11 months or ≥ 40 breaths/min for children 12-59 months) | +1 | (Age 2-11 months AND respiratory rate ≥ 70 breaths/min)  OR  (Age 12-59 months AND respiratory rate ≥ 60 breaths/min) |
| Unconscious/decreased consciousness | +1 | Conscious level = “Prostrate”  OR  Conscious level = “Unconscious” |
| Convulsions | +2 | Convulsions = “yes” |
| Cyanosis | +2 | Cyanosis = “yes” |
| Oxygen saturations < 90% | +2 | Oxygen saturations < 90% |
| **RISC (HIV-Negative)** |  |  |
| Oxygen saturation <90% | +3 | Oxygen saturation <90% |
| Oxygen saturation ≥ 90% AND chest indrawing | +2 | Oxygen saturation ≥ 90%  AND  Chest indrawing = “yes” |
| Wheezing | -2 | Wheeze = “yes” |
| Refusing feeds | +1 | Unable to drink = “yes” |
| Weight for age z-score ≤ -2 AND > -3 | +1 | Weight for age z-score ≤ -2 AND > -3 |
| Weight for age z-score ≤ -3 | +2 | Weight for age z-score ≤ -2 AND > -3 |
| **RISC-Malawi (MUAC)** |  |  |
| Moderate hypoxemia (Oxygen saturations: 90-92%) | +2 | Oxygen saturations 90-92% |
| Severe hypoxemia (Oxygen saturations <90%) | +7 | Oxygen saturations < 90% |
| Moderately malnourished (MUAC: 11.5-13.5cm) | +3 | MUAC 11.5cm – 13.5cm |
| Severely malnourished (MUAC <11.5cm) | +7 | MUAC < 11.5cm |
| Female | +1 | Sex = “Female” |
| Wheeze present | -2 | Wheeze = “yes” |
| Unconscious | +8 | Conscious level = “prostrate”  OR  Conscious level = “Unconscious” |
| **RISC-Malawi (WAZ)** |  |  |
| Moderate hypoxemia (Oxygen saturations: 90-92%) | +1 | Oxygen saturations 90-92% |
| Severe hypoxemia (Oxygen saturations <90%) | +5 | Oxygen saturations < 90% |
| Moderately malnourished (WAZ < -2 and ≥ -3) | +3 | WAZ < - 2 and ≥ -3 |
| Severely malnourished (WAZ <-3) | +6 | WAZ < -3 |
| Female | +1 | Sex = “Female” |
| Wheeze present | -1 | Wheeze = “yes” |
| Unconscious | +5 | Conscious level = “prostrate”  OR  Conscious level = “Unconscious” |

WAZ: Weight-for-age z-score; MUAC: Mid-upper arm circumference

Supplementary table 4: Inclusion criteria for main cohort (cohort A) and all cohorts used for sensitivity analysis (cohorts B-F).

| **Cohort** | **Inclusion criteria** | **Cohort size** | **Number of deaths (Case fatality rate)** |
| --- | --- | --- | --- |
|  |  | **N** | **N (%)** |
| **A** | Children 2-24 months who meet the criteria for severe ALRI on admission and have: a discharge diagnosis of ALRI, no missing data for any evaluated score component and an outcome of death or discharge | 2182 | 152 (7.0%) |
|  | | | |
| **Cohort** | **Difference from Cohort A** | **Cohort size** | **Number of deaths (Case fatality rate)** |
| B | Includes children who do not have a discharge diagnosis of ALRI | 2816 | 281 (10.0%) |
| C | Includes children up to 59 months | 2516 | 163 (6.5%) |
| D | Includes children who are missing data for evaluated score components | 2242 | 156 (7.0%) |
| E | Removes admissions which began during the year after the first case of COVID was detected in Kenya (i.e. 13^th^ March 2020 – 13^th^ March 2021 (inclusive)) | 2113 | 139 (6.6%) |
| F | Removes admissions which began after the first case of COVID was detected in Kenya (i.e. removes admissions on or after 13^th^ March 2020) | 1326 | 75 (5.7%) |

Supplementary table 5: P-value matrix from pairwise AUROC testing of scores evaluated in cohort A.

|  | **mRISC** | **PERCH** | **PREPARE** | **ReSVinet** | **ReSVinet + Nutrition (MUAC)** | **RISC** | **RISC-Malawi (MUAC)** | **RISC-Malawi (WAZ)** |
| --- | --- | --- | --- | --- | --- | --- | --- | --- |
| **mRISC** |  |  |  |  |  |  |  |  |
| **PERCH** | 0.658 |  |  |  |  |  |  |  |
| **PREPARE** | 0.211 | 0.406 |  |  |  |  |  |  |
| **ReSVinet** | 0.091 | 0.037 | 0.014 |  |  |  |  |  |
| **ReSVinet + Nutrition (MUAC)** | 0.127 | 0.286 | 0.706 | <0.001 |  |  |  |  |
| **RISC** | 0.028 | 0.006 | <0.001 | 0.647 | <0.001 |  |  |  |
| **RISC-Malawi (MUAC)** | 0.002 | <0.001 | 0.007 | <0.001 | 0.109 | <0.001 |  |  |
| **RISC-Malawi (WAZ)** | 0.496 | 0.713 | 0.486 | 0.027 | 0.427 | <0.001 | <0.001 |  |

*Results shown to 3 decimal places.*

Supplementary table 6: Score distribution for the mRISC score and sensitivity/specificity at each cut-off.

| **Score value** | **Patients assigned score**  **(n)** | **Number of deaths**  **(n)** | **Case fatality rate**  **(%)** | **Sensitivity at cut-off ≥ score**  **(%)** | **Specificity at cut-off ≥ score**  **(%)** | **Percentage over cut-off**  **(%)** | **Youden’s J index** | **Case fatality rate for ≥ score**  **(%)** |
| --- | --- | --- | --- | --- | --- | --- | --- | --- |
| 0 | 6 | 1 | 16.7 | 100.0 | 0.0 | 100.0 | 0.000 | 7.0 |
| 1 | 492 | 4 | 0.8 | 99.3 | 0.2 | 99.7 | -0.005 | 6.9 |
| 2 | 462 | 19 | 4.1 | 96.7 | 24.3 | 77.2 | 0.210 | 8.7 |
| 3 | 685 | 34 | 5.0 | 84.2 | 46.1 | 56.0 | 0.303 | 10.5 |
| 4 | 372 | 39 | 10.5 | 61.8 | 78.2 | 24.6 | 0.400 | 17.5 |
| 5 | 128 | 38 | 29.7 | 36.2 | 94.6 | 7.6 | 0.308 | 33.3 |
| 6 | 32 | 13 | 40.6 | 11.2 | 99.0 | 1.7 | 0.102 | 45.9 |
| 7 | 5 | 4 | 80.0 | 2.6 | 100.0 | 0.2 | 0.026 | 80.0 |

Supplementary table 7: Score distribution for the PERCH score and sensitivity/specificity at each cut-off.

| **Score value** | **Patients assigned score**  **(n)** | **Number of deaths**  **(n)** | **Case fatality rate**  **(%)** | **Sensitivity at cut-off ≥ score**  **(%)** | **Specificity at cut-off ≥ score**  **(%)** | **Percentage over cut-off**  **(%)** | **Youden’s J index** | **Case fatality rate for ≥ score**  **(%)** |
| --- | --- | --- | --- | --- | --- | --- | --- | --- |
| -1 | 165 | 1 | 0.6 | 100.0 | 0.0 | 100.0 | 0.000 | 7.0 |
| 0 | 134 | 3 | 2.2 | 99.3 | 8.1 | 92.4 | 0.074 | 7.5 |
| 1 | 714 | 20 | 2.8 | 97.4 | 14.5 | 86.3 | 0.119 | 7.9 |
| 2 | 545 | 19 | 3.5 | 84.2 | 48.7 | 53.6 | 0.329 | 10.9 |
| 3 | 219 | 25 | 11.4 | 71.7 | 74.6 | 28.6 | 0.463 | 17.5 |
| 4 | 194 | 26 | 13.4 | 55.3 | 84.2 | 18.6 | 0.395 | 20.7 |
| 5 | 66 | 21 | 31.8 | 38.2 | 92.5 | 9.7 | 0.307 | 27.5 |
| 6 | 58 | 10 | 17.2 | 24.3 | 94.7 | 6.6 | 0.190 | 25.5 |
| 7 | 28 | 9 | 32.1 | 17.8 | 97.0 | 4.0 | 0.148 | 31.0 |
| 8 | 20 | 2 | 10.0 | 11.8 | 98.0 | 2.7 | 0.098 | 30.5 |
| 9 | 25 | 10 | 40.0 | 10.5 | 98.9 | 1.8 | 0.094 | 41.0 |
| 10 | 3 | 1 | 33.3 | 3.9 | 99.6 | 0.6 | 0.035 | 42.9 |
| 11 | 8 | 4 | 50.0 | 3.3 | 99.7 | 0.5 | 0.030 | 45.5 |
| 12 | 3 | 1 | 33.3 | 0.7 | 99.9 | 0.1 | 0.006 | 33.3 |

Supplementary table 8: Score distribution for the PREPARE score and sensitivity/specificity at each cut-off.

| **Score value** | **Patients assigned score**  **(n)** | **Number of deaths**  **(n)** | **Case fatality rate**  **(%)** | **Sensitivity at cut-off ≥ score**  **(%)** | **Specificity at cut-off ≥ score**  **(%)** | **Percentage over cut-off**  **(%)** | **Youden’s J index** | **Case fatality rate for ≥ score**  **(%)** |
| --- | --- | --- | --- | --- | --- | --- | --- | --- |
| 0 | 96 | 0 | 0.0 | 100.0 | 0.0 | 100.0 | 0.000 | 7.0 |
| 1 | 289 | 6 | 2.1 | 100.0 | 4.7 | 95.6 | 0.047 | 7.3 |
| 2 | 537 | 12 | 2.2 | 96.1 | 18.7 | 82.4 | 0.148 | 8.1 |
| 3 | 475 | 19 | 4.0 | 88.2 | 44.5 | 57.7 | 0.327 | 10.6 |
| 4 | 330 | 19 | 5.8 | 75.7 | 67.0 | 36.0 | 0.427 | 14.6 |
| 5 | 200 | 25 | 12.5 | 63.2 | 82.3 | 20.9 | 0.455 | 21.1 |
| 6 | 129 | 29 | 22.5 | 46.7 | 90.9 | 11.7 | 0.376 | 27.8 |
| 7 | 67 | 17 | 25.4 | 27.6 | 95.9 | 5.8 | 0.235 | 33.3 |
| 8 | 34 | 10 | 29.4 | 16.4 | 98.3 | 2.7 | 0.147 | 42.4 |
| 9 | 19 | 12 | 63.2 | 9.9 | 99.5 | 1.1 | 0.094 | 60.0 |
| 10 | 5 | 3 | 60.0 | 2.0 | 99.9 | 0.3 | 0.019 | 50.0 |
| 11 | 1 | 0 | 0.0 | 0.0 | 100.0 | 0.0 | 0.000 | 0.0 |

Supplementary table 9: Score distribution for the ReSVinet score and sensitivity/specificity at each cut-off.

| **Score value** | **Patients assigned score**  **(n)** | **Number of deaths**  **(n)** | **Case fatality rate**  **(%)** | **Sensitivity at cut-off ≥ score**  **(%)** | **Specificity at cut-off ≥ score**  **(%)** | **Percentage over cut-off**  **(%)** | **Youden’s J index** | **Case fatality rate for ≥ score**  **(%)** |
| --- | --- | --- | --- | --- | --- | --- | --- | --- |
| 2 | 81 | 2 | 2.5 | 100.0 | 0.0 | 100.0 | 0.000 | 7.0 |
| 3 | 42 | 0 | 0.0 | 98.7 | 3.9 | 96.3 | 0.026 | 7.1 |
| 4 | 265 | 9 | 3.4 | 98.7 | 6.0 | 94.4 | 0.047 | 7.3 |
| 5 | 284 | 10 | 3.5 | 92.8 | 18.6 | 82.2 | 0.114 | 7.9 |
| 6 | 434 | 15 | 3.5 | 86.2 | 32.1 | 69.2 | 0.183 | 8.7 |
| 7 | 283 | 13 | 4.6 | 76.3 | 52.7 | 49.3 | 0.290 | 10.8 |
| 8 | 254 | 17 | 6.7 | 67.8 | 66.0 | 36.3 | 0.338 | 13.0 |
| 9 | 188 | 16 | 8.5 | 56.6 | 77.7 | 24.7 | 0.343 | 16.0 |
| 10 | 142 | 20 | 14.1 | 46.1 | 86.2 | 16.1 | 0.323 | 19.9 |
| 11 | 91 | 19 | 20.9 | 32.9 | 92.2 | 9.6 | 0.251 | 23.9 |
| 12 | 51 | 10 | 19.6 | 20.4 | 95.7 | 5.4 | 0.161 | 26.3 |
| 13 | 52 | 17 | 32.7 | 13.8 | 97.7 | 3.1 | 0.115 | 31.3 |
| 14 | 15 | 4 | 26.7 | 2.6 | 99.5 | 0.7 | 0.021 | 26.7 |

Supplementary table 10: Score distribution for RISC (HIV-Negative) score and sensitivity/specificity at each cut-off.

| **Score value** | **Patients assigned score**  **(n)** | **Number of deaths**  **(n)** | | **Case fatality rate**  **(%)** | **Sensitivity at cut-off ≥ score**  **(%)** | **Specificity at cut-off ≥ score**  **(%)** | **Percentage over cut-off**  **(%)** | **Youden’s J index** | **Case fatality rate for ≥ score**  **(%)** |
| --- | --- | --- | --- | --- | --- | --- | --- | --- | --- |
| -1 | 1 | 0 | 0.0 | | 100.0 | 0.0 | 100.0 | 0.000 | 7.0 |
| 0 | 239 | 6 | 2.5 | | 100.0 | 0.0 | 100.0 | 0.000 | 7.0 |
| 1 | 93 | 4 | 4.3 | | 96.1 | 11.5 | 89.0 | 0.076 | 7.5 |
| 2 | 906 | 32 | 3.5 | | 93.4 | 15.9 | 84.7 | 0.093 | 7.7 |
| 3 | 478 | 36 | 7.5 | | 72.4 | 59.0 | 43.2 | 0.314 | 11.7 |
| 4 | 361 | 39 | 10.8 | | 48.7 | 80.7 | 21.3 | 0.294 | 15.9 |
| 5 | 92 | 29 | 31.5 | | 23.0 | 96.6 | 4.8 | 0.196 | 33.7 |
| 6 | 12 | 6 | 50.0 | | 3.9 | 99.7 | 0.5 | 0.036 | 50.0 |

Supplementary table 11: Score distribution for RISC-Malawi (MUAC) score and sensitivity/specificity at each cut-off.

| **Score value** | **Patients assigned score**  **(n)** | **Number of deaths**  **(n)** | **Case fatality rate**  **(%)** | **Sensitivity at cut-off ≥ score**  **(%)** | **Specificity at cut-off ≥ score**  **(%)** | **Percentage over cut-off**  **(%)** | **Youden’s J index** | **Case fatality rate for ≥ score**  **(%)** |
| --- | --- | --- | --- | --- | --- | --- | --- | --- |
| -2 | 99 | 3 | 3.0 | 100.0 | 0.0 | 100.0 | 0.000 | 7.0 |
| -1 | 44 | 0 | 0.0 | 98.0 | 4.7 | 95.5 | 0.027 | 7.2 |
| 0 | 286 | 0 | 0.0 | 98.0 | 6.9 | 93.4 | 0.049 | 7.3 |
| 1 | 216 | 4 | 1.9 | 98.0 | 21.0 | 80.3 | 0.190 | 8.5 |
| 2 | 64 | 1 | 1.6 | 95.4 | 31.4 | 70.4 | 0.268 | 9.4 |
| 3 | 388 | 10 | 2.6 | 94.7 | 34.5 | 67.5 | 0.292 | 9.8 |
| 4 | 312 | 6 | 1.9 | 88.2 | 53.2 | 49.7 | 0.414 | 12.4 |
| 5 | 34 | 1 | 2.9 | 84.2 | 68.2 | 35.4 | 0.524 | 16.6 |
| 6 | 35 | 2 | 5.7 | 83.6 | 69.9 | 33.9 | 0.535 | 17.2 |
| 7 | 171 | 14 | 8.2 | 82.2 | 71.5 | 32.3 | 0.537 | 17.8 |
| 8 | 169 | 16 | 9.5 | 73.0 | 79.2 | 24.4 | 0.522 | 20.8 |
| 9 | 28 | 3 | 10.7 | 62.5 | 86.7 | 16.7 | 0.492 | 26.1 |
| 10 | 57 | 13 | 22.8 | 60.5 | 88.0 | 15.4 | 0.485 | 27.4 |
| 11 | 72 | 12 | 16.7 | 52.0 | 90.1 | 12.8 | 0.421 | 28.3 |
| 12 | 21 | 6 | 28.6 | 44.1 | 93.1 | 9.5 | 0.372 | 32.4 |
| 13 | 18 | 4 | 22.2 | 40.1 | 93.8 | 8.5 | 0.339 | 32.8 |
| 14 | 37 | 16 | 43.2 | 37.5 | 94.5 | 7.7 | 0.320 | 33.9 |
| 15 | 53 | 11 | 20.8 | 27.0 | 95.6 | 6.0 | 0.226 | 31.3 |
| 16 | 19 | 11 | 57.9 | 19.7 | 97.6 | 3.6 | 0.173 | 38.5 |
| 17 | 5 | 0 | 0.0 | 12.5 | 98.0 | 2.7 | 0.105 | 32.2 |
| 18 | 18 | 3 | 16.7 | 12.5 | 98.3 | 2.5 | 0.108 | 35.2 |
| 19 | 8 | 3 | 37.5 | 10.5 | 99.0 | 1.6 | 0.095 | 44.4 |
| 21 | 1 | 1 | 100.0 | 8.6 | 99.3 | 1.3 | 0.079 | 46.4 |
| 22 | 12 | 7 | 58.3 | 7.9 | 99.3 | 1.2 | 0.072 | 44.4 |
| 23 | 15 | 5 | 33.3 | 3.3 | 99.5 | 0.7 | 0.028 | 33.3 |

Supplementary table 12: Score distribution for RISC-Malawi (WAZ) score and sensitivity/specificity at each cut-off.

| **Score value** | **Patients assigned score**  **(n)** | **Number of deaths**  **(n)** | **Case fatality rate**  **(%)** | **Sensitivity at cut-off ≥ score**  **(%)** | **Specificity at cut-off ≥ score**  **(%)** | **Percentage over cut-off**  **(%)** | **Youden’s J index** | **Case fatality rate for ≥ score**  **(%)** |
| --- | --- | --- | --- | --- | --- | --- | --- | --- |
| -1 | 164 | 5 | 3.0 | 100.0 | 0.0 | 100.0 | 0.000 | 7.0 |
| 0 | 600 | 10 | 1.7 | 96.7 | 7.8 | 92.5 | 0.045 | 7.3 |
| 1 | 397 | 11 | 2.8 | 90.1 | 36.9 | 65.0 | 0.270 | 9.7 |
| 2 | 43 | 2 | 4.7 | 82.9 | 55.9 | 46.8 | 0.388 | 12.3 |
| 3 | 171 | 7 | 4.1 | 81.6 | 57.9 | 44.8 | 0.395 | 12.7 |
| 4 | 147 | 6 | 4.1 | 77.0 | 66.0 | 37.0 | 0.430 | 14.5 |
| 5 | 124 | 15 | 12.1 | 73.0 | 73.0 | 30.2 | 0.460 | 16.8 |
| 6 | 194 | 19 | 9.8 | 63.2 | 78.3 | 24.6 | 0.415 | 17.9 |
| 7 | 111 | 9 | 8.1 | 50.7 | 86.9 | 15.7 | 0.376 | 22.5 |
| 8 | 44 | 13 | 29.5 | 44.7 | 92.0 | 10.6 | 0.367 | 29.4 |
| 9 | 33 | 6 | 18.2 | 36.2 | 93.5 | 8.6 | 0.297 | 29.4 |
| 10 | 23 | 3 | 13.0 | 32.2 | 94.8 | 7.1 | 0.270 | 31.8 |
| 11 | 49 | 15 | 30.6 | 30.3 | 95.8 | 6.0 | 0.261 | 35.1 |
| 12 | 52 | 16 | 30.8 | 20.4 | 97.5 | 3.8 | 0.179 | 37.8 |
| 13 | 8 | 4 | 50.0 | 9.9 | 99.3 | 1.4 | 0.092 | 50.0 |
| 14 | 9 | 3 | 33.3 | 7.2 | 99.5 | 1.0 | 0.067 | 50.0 |
| 16 | 9 | 6 | 66.7 | 5.3 | 99.8 | 0.6 | 0.051 | 61.5 |
| 17 | 4 | 2 | 50.0 | 1.3 | 99.9 | 0.2 | 0.012 | 50.0 |

Supplementary table 13: AUROCs for in-hospital mortality, stratified by age group.

| **Score** | **AUROC for in-hospital mortality (95% confidence interval)** | | |
| --- | --- | --- | --- |
|  | **2-5 months**  **(n = 863)** | **6-12 months**  **(n = 766)** | **13-24 months**  **(n = 553)** |
| mRISC | 0.69 (0.63 - 0.75) | 0.79 (0.72 - 0.85) | 0.77 (0.66 - 0.88) |
| PERCH | 0.73 (0.67 - 0.79) | 0.78 (0.7 - 0.85) | 0.85 (0.76 - 0.93) |
| PREPARE | 0.77 (0.71 - 0.82) | 0.8 (0.74 - 0.87) | 0.77 (0.67 - 0.87) |
| ReSVinet | 0.69 (0.62 - 0.76) | 0.75 (0.68 - 0.82) | 0.8 (0.71 - 0.9) |
| ReSVinet + Nutrition (MUAC) | 0.77 (0.71 - 0.82) | 0.81 (0.75 - 0.86) | 0.85 (0.77 - 0.93) |
| RISC (HIV-Negative) | 0.73 (0.67 - 0.79) | 0.72 (0.63 - 0.81) | 0.68 (0.58 - 0.79) |
| RISC-Malawi (MUAC) | 0.81 (0.76 - 0.86) | 0.79 (0.72 - 0.87) | 0.87 (0.78 - 0.95) |
| RISC-Malawi (WAZ) | 0.77 (0.71 - 0.83) | 0.81 (0.74 - 0.89) | 0.81 (0.72 - 0.9) |

Supplementary table 14: p-values of unpaired AUROC tests for age stratification.

| **score** | **p-values of unpaired ROC tests** | | |
| --- | --- | --- | --- |
|  | **2-5 months vs. 6-12 months** | **2-5 months vs. 13-24 months** | **6-12 months vs. 13-24 months** |
| mRISC | 0.045 | 0.232 | 0.820 |
| PERCH | 0.331 | 0.024 | 0.222 |
| PREPARE | 0.390 | 0.936 | 0.589 |
| ReSVinet | 0.228 | 0.054 | 0.354 |
| ReSVinet + Nutrition (MUAC) | 0.300 | 0.076 | 0.362 |
| RISC (HIV-Negative) | 0.867 | 0.450 | 0.599 |
| RISC-Malawi (MUAC) | 0.712 | 0.234 | 0.201 |
| RISC-Malawi (WAZ) | 0.368 | 0.433 | 0.989 |

Supplementary table 15: AUROCs for in-hospital mortality, for scores assessed in all admissions 2-24 months regardless of discharge diagnosis (Cohort B)

| **Score** | **AUROC for in-hospital mortality**  **(95% CI)** | **p-value of test for difference with Cohort A AUROCs** |
| --- | --- | --- |
| mRISC | 0.75 (0.72 - 0.78) | 0.654 |
| PERCH | 0.76 (0.73 - 0.79) | 0.789 |
| PREPARE | 0.74 (0.71 - 0.77) | 0.102 |
| ReSVinet | 0.71 (0.68 - 0.75) | 0.883 |
| ReSVinet + Nutrition (MUAC) | 0.77 (0.74 - 0.80) | 0.318 |
| RISC (HIV-Negative) | 0.66 (0.63 - 0.70) | 0.159 |
| RISC-Malawi (MUAC) | 0.80 (0.77 - 0.83) | 0.254 |
| RISC-Malawi (WAZ) | 0.77 (0.74 - 0.80) | 0.686 |

Supplementary table 16: AUROCs for in-hospital mortality, for scores assessed in admissions for children 2-59 months with a primary or secondary discharge diagnosis of ALRI (Cohort C)

| **Score** | **AUROC for in-hospital mortality**  **(95% CI)** | **p-value of test for difference with Cohort A AUROCs** |
| --- | --- | --- |
| mRISC | 0.78 (0.74 - 0.81) | 0.615 |
| PERCH | 0.77 (0.74 - 0.81) | 0.918 |
| PREPARE | 0.79 (0.75 - 0.83) | 0.888 |
| RISC | 0.71 (0.66 - 0.75) | 0.923 |
| RISC-Malawi (MUAC) | 0.83 (0.80 - 0.87) | 0.763 |
| RISC-Malawi (WAZ) | 0.78 (0.74 - 0.82) | 0.896 |

*ReSVinet was not included in this sensitivity analysis, as it only provides respiratory rate thresholds for children under 36 months.

Supplementary table 17: AUROCs for in-hospital mortality, including children with missing data (Cohort D)

| **Score** | **AUROC for in-hospital mortality (95% CI)** | **p-value of test for difference with cohort A AUROCs** |
| --- | --- | --- |
| mRISC | 0.76 (0.72 - 0.8) | 0.868 |
| PERCH | 0.76 (0.72 - 0.8) | 0.772 |
| PREPARE | 0.78 (0.75 - 0.82) | 0.955 |
| ReSVinet | 0.7 (0.66 - 0.74) | 0.568 |
| ReSVinet + Nutrition (MUAC) | 0.78 (0.74 - 0.81) | 0.493 |
| RISC (HIV-Negative) | 0.75 (0.7 - 0.79) | 0.172 |
| RISC-Malawi (MUAC) | 0.82 (0.79 - 0.86) | 0.970 |
| RISC-Malawi (WAZ) | 0.78 (0.73 - 0.82) | 0.959 |

Supplementary table 18: AUROCs for in-hospital mortality with admissions occurring in the year following the first case of COVID-19 excluded (i.e. Patients admitted between 13th March 2020 and 13th March 2021 (inclusive) not included). (Cohort E)

| **Score** | **AUROC for in-hospital mortality (95% confidence interval)**  **(n = 2114)** | **p-value of test for difference with cohort A AUROCs** |
| --- | --- | --- |
| mRISC | 0.76 (0.72 - 0.80) | 0.971 |
| PERCH | 0.78 (0.74 - 0.82) | 0.746 |
| PREPARE | 0.80 (0.76 - 0.84) | 0.589 |
| ReSVinet | 0.72 (0.67 - 0.77) | 0.922 |
| ReSVinet + Nutrition (MUAC) | 0.80 (0.76 - 0.84) | 0.819 |
| RISC (HIV-Negative) | 0.72 (0.67 - 0.76) | 0.711 |
| RISC-Malawi (MUAC) | 0.84 (0.81 - 0.87) | 0.581 |
| RISC-Malawi (WAZ) | 0.79 (0.75 - 0.83) | 0.672 |

Supplementary table 19: AUROCs for in-hospital mortality, excluding admissions post-COVID (i.e. excluding admissions on or after 13/03/20). (Cohort F)

| **Score** | **AUROC for in-hospital mortality (95% confidence interval)**  **(n = 1325)** | **p-value of test for difference with cohort A AUROCs** |
| --- | --- | --- |
| mRISC | 0.78 (0.72 - 0.83) | 0.655 |
| PERCH | 0.79 (0.74 - 0.84) | 0.608 |
| PREPARE | 0.84 (0.79 - 0.88) | 0.089 |
| ReSVinet | 0.73 (0.66 - 0.79) | 0.849 |
| ReSVinet + Nutrition (MUAC) | 0.81 (0.76 - 0.86) | 0.569 |
| RISC (HIV-Negative) | 0.74 (0.68 - 0.8) | 0.364 |
| RISC-Malawi (MUAC) | 0.85 (0.8 - 0.89) | 0.420 |
| RISC-Malawi (WAZ) | 0.82 (0.77 - 0.87) | 0.236 |

Supplementary table 20: AUROCs for in-hospital mortality for modified ReSVinet score, using either mid-upper arm circumference, weight-for-length z-score or weight-for-age z-score as the malnutrition indicator.

| **Score** | **AUROCs for in-hospital mortality**  **(95% CI)** |
| --- | --- |
| ReSVinet + Nutrition (MUAC) | 0.79 (0.76 – 0.83) |
| ReSVinet + Nutrition (WAZ) | 0.75 (0.71 – 0.79) |
| ReSVinet + Nutrition (WLZ) | 0.73 (0.68 – 0.77) |

Supplementary table 21: P-value matrix from pairwise testing of modified ReSVinet scores.

|  | **ReSVinet** | **ReSVinet + Nutrition (MUAC)** | **ReSVinet + Nutrition (WAZ)** | **ReSVinet + Nutrition (WLZ)** |
| --- | --- | --- | --- | --- |
| **ReSVinet** |  |  |  |  |
| **ReSVinet + Nutrition (MUAC)** | <0.001 |  |  |  |
| **ReSVinet + Nutrition (WAZ)** | 0.001 | <0.001 |  |  |
| **ReSVinet + Nutrition (WLZ)** | 0.291 | <0.001 | 0.009 |  |

Supplementary table 22: Score distribution for the ReSVinet + Nutrition (MUAC) score and sensitivity/specificity at each cut-off.

| **Score value** | **Patients assigned score**  **(n)** | **Number of deaths**  **(n)** | **Case fatality rate**  **(%)** | **Sensitivity at cut-off ≥ score**  **(%)** | **Specificity at cut-off ≥ score**  **(%)** | **Percentage over cut-off**  **(%)** | **Youden’s J index** | **Case fatality rate for ≥ score**  **(%)** |
| --- | --- | --- | --- | --- | --- | --- | --- | --- |
| 2 | 50 | 0 | 0.0 | 100.0 | 0.0 | 100.0 | 0.000 | 7.0 |
| 3 | 25 | 0 | 0.0 | 100.0 | 2.5 | 97.7 | 0.025 | 7.1 |
| 4 | 180 | 0 | 0.0 | 100.0 | 3.7 | 96.6 | 0.037 | 7.2 |
| 5 | 198 | 2 | 1.0 | 100.0 | 12.6 | 88.3 | 0.126 | 7.9 |
| 6 | 313 | 5 | 1.6 | 98.7 | 22.2 | 79.2 | 0.209 | 8.7 |
| 7 | 283 | 14 | 4.9 | 95.4 | 37.4 | 64.9 | 0.328 | 10.2 |
| 8 | 299 | 15 | 5.0 | 86.2 | 50.6 | 51.9 | 0.368 | 11.6 |
| 9 | 257 | 16 | 6.2 | 76.3 | 64.6 | 38.2 | 0.409 | 13.9 |
| 10 | 179 | 15 | 8.4 | 65.8 | 76.5 | 26.4 | 0.423 | 17.3 |
| 11 | 142 | 17 | 12.0 | 55.9 | 84.6 | 18.2 | 0.405 | 21.4 |
| 12 | 91 | 14 | 15.4 | 44.7 | 90.7 | 11.7 | 0.354 | 26.6 |
| 13 | 87 | 26 | 29.9 | 35.5 | 94.5 | 7.6 | 0.300 | 32.7 |
| 14 | 32 | 8 | 25.0 | 18.4 | 97.5 | 3.6 | 0.159 | 35.9 |
| 15 | 22 | 8 | 36.4 | 13.2 | 98.7 | 2.1 | 0.119 | 43.5 |
| 16 | 17 | 8 | 47.1 | 7.9 | 99.4 | 1.1 | 0.073 | 50.0 |
| 17 | 7 | 4 | 57.1 | 2.6 | 99.9 | 0.3 | 0.025 | 57.1 |

**References**

1. Justicia-Grande AJ, Pardo-Seco J, Cebey-López M, et al. Development and Validation of a New Clinical Scale for Infants with Acute Respiratory Infection: The ReSVinet Scale. Esposito S, editor. PLoS ONE. **2016**; 11(6):e0157665.

2. Reed C, Madhi SA, Klugman KP, et al. Development of the Respiratory Index of Severity in Children (RISC) Score among Young Children with Respiratory Infections in South Africa. Jhaveri R, editor. PLoS ONE. **2012**; 7(1):e27793.

3. Emukule GO, McMorrow M, Ulloa C, et al. Predicting Mortality among Hospitalized Children with Respiratory Illness in Western Kenya, 2009–2012. Metcalfe JZ, editor. PLoS ONE. **2014**; 9(3):e92968.

4. Hooli S, Colbourn T, Lufesi N, et al. Predicting Hospitalised Paediatric Pneumonia Mortality Risk: An External Validation of RISC and mRISC, and Local Tool Development (RISC-Malawi) from Malawi. PLOS ONE. **2016**; 11(12):e0168126.

5. Gallagher KE, Knoll MD, Prosperi C, et al. The Predictive Performance of a Pneumonia Severity Score in Human Immunodeficiency Virus–negative Children Presenting to Hospital in 7 Low- and Middle-income Countries. Clinical Infectious Diseases. **2020**; 70(6):1050–1057.

6. Rees CA, Colbourn T, Hooli S, et al. Derivation and validation of a novel risk assessment tool to identify children aged 2–59 months at risk of hospitalised pneumonia-related mortality in 20 countries. BMJ Glob Health. **2022**; 7(4):e008143.
